# Outcomes of Retinopathy of Prematurity

**Published:** 2016

**Authors:** Fatemeh HEIDARY, Reza GHAREBAGHI

**Affiliations:** 1 Immunoregulation Research Center, Shahed University, Tehran, Iran; 2 International Virtual Ophthalmic Research Center (IVORC)

**Keywords:** Complication, Retinopathy of Prematurity, Blindness, Refractive Errors, Strabismus, Visual Impairment

Growing rates of premature birth have led to increases in the prevalence of retinopathy of prematurity (ROP), which is now becoming a leading cause of childhood blindness worldwide (1). At the same time, cost-effective and accessible screening methods and medical services may reduce its incidence. Prompt approaches to management of complications, as well as providing low vision aids for children with residual visual capacity, may be useful approaches in managing ROP (2). Individuals with ROP require lifelong ophthalmology follow-up, as premature children with useful vision may develop ocular morbidities in later life. These morbidities include myopia, anisometropia, traction of retina, retinal detachment, strabismus, and amblyopia. Furthermore, impairments in visual acuity, contrast sensitivity, visual field, convergence, and accommodation may be discovered during follow-up (3).

In their population-based study, Darlow and associates found that very low birth weight was associated with a higher risk for compromised vision during school age. This study indicated that a history of ROP is associated with poorer vision and an almost two-fold worsening of distance acuity, strabismus, and myopia (4). A 20% rise in the prevalence of strabismus was noted in low birth weight children. This increase was associated with the stage of ROP. Specifically, the incidence of strabismus was 6% in stage 1 ROP and reached more than 30% in stage 3 ROP. However, even in eyes with poor visual performance, early treatment led to improvements in vision (5). In their prospective study Stephenson and colleagues examined survivors of low birth weight (<1,701 g) born between 1985 and 1987 at the ages of 10-13 years. They found that 50% of study subjects had adverse ophthalmic outcomes, which were associated with poor cognitive results at 11-14 years of age (6). 

A recent and unique study by Hellgren et al. assessed vision in a national cohort of extremely pretermature infants at the age of 6.5 years. The 486 study subjects had significantly higher rates of blindness, refractive errors, strabismus, and visual impairment based on World Health Organization (WHO) criteria. However, the authors did not find an association between visual impairment and gestational age following adjustments for ROP that required treatment (7). 

In another cohort study, Smith et al. reported that significant late complications of ROP in infants born between 1946 and 1964 included ROP-related posterior segment complications, such as retinal detachment, cataract, and myopia. In this study, the most important outcome measure was best-corrected visual acuity. Specifically, 51.2% of study subjects had visual acuity of ≤20/200 and 41.7% had visual acuity of ≥20/60 (8). 

Previous studies have emphasized the long-term visual impact of ROP and its serious visual morbidities. However, in most communities, health care workers, educators, and patients’ relatives are not familiar with visual impairments in these children. Indeed, programs to enhance awareness of ROP in the community may lead to collaborations aimed at engaging visually disabled children in educational programs. In addition, the establishment of special schools with auxiliary educational assistance and skilled education teams are essential for children who are unable to study in ordinary schools due to multiple disabilities. Hiring qualified teachers in these special schools and supplying them with auxiliary educational aids may enhance the abilities of visually impaired children. In fact, the use of trained teachers and the appropriate teaching materials may give these students the chance to study in local schools. These students should be taught how to use specialized learning materials and auxiliary educational aids (9). Disorders of visual perception, including depth perception, orientation, simultaneous perception, perception of movement, and recognition caused by ocular diseases or brain disorders have become more prevalent in prematurely born children. Therefore, neurologic assessment of these children seems to be necessary in addition to ocular examinations (4-7). 

In conclusion, ROP is still a major risk factor for blindness worldwide. Premature babies face a lifelong risk of visual disabilities and ophthalmic problems. Therefore, to lower the rates of overall complications, the importance of lifelong ophthalmology follow-ups should be emphasized to parents and guardians. In addition, public media should be used to increase awareness of ROP. Finally, the establishment of ROP teams consisting of individuals with related medical subspecialties in tertiary referral centers seems worthwhile and effective.
